# Genomic Characterization of Imipenem- and Imipenem-Relebactam-Resistant Clinical Isolates of Pseudomonas aeruginosa

**DOI:** 10.1128/mSphere.00836-21

**Published:** 2021-11-24

**Authors:** Mario López-Pérez, Jose M. Haro-Moreno, Carmen Molina-Pardines, Maria Paz Ventero, Juan Carlos Rodríguez

**Affiliations:** a Evolutionary Genomics Group, División de Microbiología, Universidad Miguel Hernández, Alicante, Spain; b Microbiology Department, Alicante University General Hospital, Alicante Institute of Sanitary and Biomedical Research (ISABIAL), Alicante, Spain; U.S. Department of Energy Joint Genome Institute

**Keywords:** *Pseudomonas aeruginosa*, imipenem, carbapenem resistance, integrative and conjugative element, antibiotic resistance, imipenem-relebactam

## Abstract

Pseudomonas aeruginosa is an opportunistic human pathogen and a major cause of nosocomial infections. The global spread of carbapenem-resistant strains is growing rapidly and has become a major public health challenge. Imipenem-relebactam (I/R) is a novel carbapenem-beta-lactamase inhibitor combination that can overcome carbapenem resistance. In this study, we aimed to understand the mechanism underlying resistance to imipenem and imipenem-relebactam. For this purpose, we performed a genomic comparison of 40 new clinical P. aeruginosa strains with different antibiotic sensitivity patterns as well as the presence/absence of carbapenemases. Results indicated the presence of a reduced flexible genome (15% total) mostly represented by phages and defense mechanisms against them, showing an important role in evolution and pathogenicity. We found a high diversity of antibiotic resistance genes grouped in small clusters mobilized via integrative and conjugative elements and facilitated by the high homologous recombination detected. Ortholog genes were found in several pathogenic strains from distantly related taxa in different mobile elements with a global distribution. The microdiversity found in those strains without carbapenemases did not reveal a clear pattern that could be associated with carbapenem resistance, suggesting multiple mechanisms of resistance in the core genome. Our results provide new insight into the dynamics and high genomic plasticity by which clinical strains of P. aeruginosa acquire resistance. This knowledge can be applied to other multidrug-resistant microbes to create predictive frameworks for assessing common molecular mechanisms of antibiotic resistance and integrated into new strategies for their prevention.

**IMPORTANCE** The growing emergence and spread of carbapenem-resistant pathogens worldwide exacerbate the clinical challenge of treating these infections. Given the importance of carbapenems for the treatment of infections caused by Pseudomonas aeruginosa, this study aimed to investigate the underlying genomic properties of the clinical isolates that exhibited resistance to imipenem and imipenem-relebactam. This information will enhance our ability to forecast traits of resistant strains and design reliable treatments against this important threat. Our results provide new insight into the dynamics and high genomic plasticity by which clinical strains of P. aeruginosa acquire resistance as well as offers a methodology that can be applied to many other opportunistic pathogens with broad antibiotic resistance.

## INTRODUCTION

Pseudomonas aeruginosa is an opportunistic human pathogen that has become a real concern in hospital-acquired infections due to its high rates of antibiotic resistance (1). Because of the difficulty of treating infections caused by this pathogen, P. aeruginosa has been classified, according to the World Health Organization, as “critical” in the global priority pathogens list of antibiotic-resistant bacteria (2).

The genome of P. aeruginosa encodes a wide variety of efflux pump systems that together with a low permeability of the outer membrane make bacteria of this species intrinsically resistant to antibiotics (1). In addition, multidrug resistance can be achieved by (i) horizontal transfer of mobile genetic elements (MGEs) carrying resistance determinants, (ii) mutation of target genes leading to gene disruption or modification of gene expression, and (iii) biofilm-mediated resistance (1, 3).

Carbapenems are commonly used in clinical practice to treat infections caused by P. aeruginosa (4). Despite its efficacy, in recent years, the rate of carbapenem resistance strains has increased worldwide (5, 6). The main mechanism of resistance is the production of carbapenemases (7, 8); however, there is a high percentage of resistant strains that do not present carbapenemases. Different mechanisms have been identified in these non-carbapenemase-producing bacteria, such as overexpression of efflux pumps, downregulation or loss of outer membrane porins, and production of ampicillin C-type β-lactamases (9).

Therefore, due to the wide genomic arsenal with which P. aeruginosa strains are endowed to face this type of antibiotic, it is necessary to develop new treatments (10). One of these novel strategies is the use of relebactam, a novel diazabicyclooctane beta-lactamase inhibitor, combined with imipenem (Im) (11). Recently, its activity has been tested *in vitro* against a large collection of multidrug-resistant clinical isolates of P. aeruginosa in several independent studies, and the prevalence of susceptible strains was always higher than 92% (11–13).

Given the importance of carbapenems for the treatment of infections caused by P. aeruginosa, this study aimed to investigate the underlying genomic properties of the clinical isolates that exhibited resistance to Im and imipenem-relebactam (I/R). Addressing these properties will enhance our ability to forecast traits of resistant strains and design reliable treatments against this important threat.

## RESULTS AND DISCUSSION

To investigate possible mechanisms underlying antibiotic resistance in P. aeruginosa, we isolated 40 new strains from different types of clinical samples (see Table S1 in the supplemental material). All strains were identified using matrix-assisted laser desorption ionization–time of flight mass spectrometry (MALDI-TOF MS) (14) and subsequently sequenced for confirmation by phylogenomic analysis, as described below. The genomic features of the strains sequenced are shown in Table S3 in the supplemental material. Based on the analysis of the resistance to beta-lactam antibiotics as well as the presence of carbapenemases (see Materials Methods) (Table S1), we were able to cluster strains into 5 groups. Strains resistant to both Im and I/R without carbapenemases were designated group 1 (G1; 6 strains) and with carbapenemases group 2 (G2; 9 strains). Group 3 (G3; 13 strains) included strains resistant to Im but sensitive to I/R without carbapenemases, and group 4 (G4; 2 strains) included those with carbapenemases. In group 5 (G5; 10 strains) were strains that were sensitive to both antibiotics.

10.1128/mSphere.00836-21.6 TABLE S1 Phenotypic characterization of antibiotic resistance. Download Table S1, XLSX file, 0.01 MB. Copyright © 2021 López-Pérez et al. 2021 López-Pérez et al. https://creativecommons.org/licenses/by/4.0/ This content is distributed under the terms of the Creative Commons Attribution 4.0 International license.

10.1128/mSphere.00836-21.8 TABLE S3 Genomic features of P. aeruginosa strains. Download Table S3, XLSX file, 0.01 MB. Copyright © 2021 López-Pérez et al. 2021 López-Pérez et al. https://creativecommons.org/licenses/by/4.0/ This content is distributed under the terms of the Creative Commons Attribution 4.0 International license.

### Phylogenomic classification.

**FIG 1:**
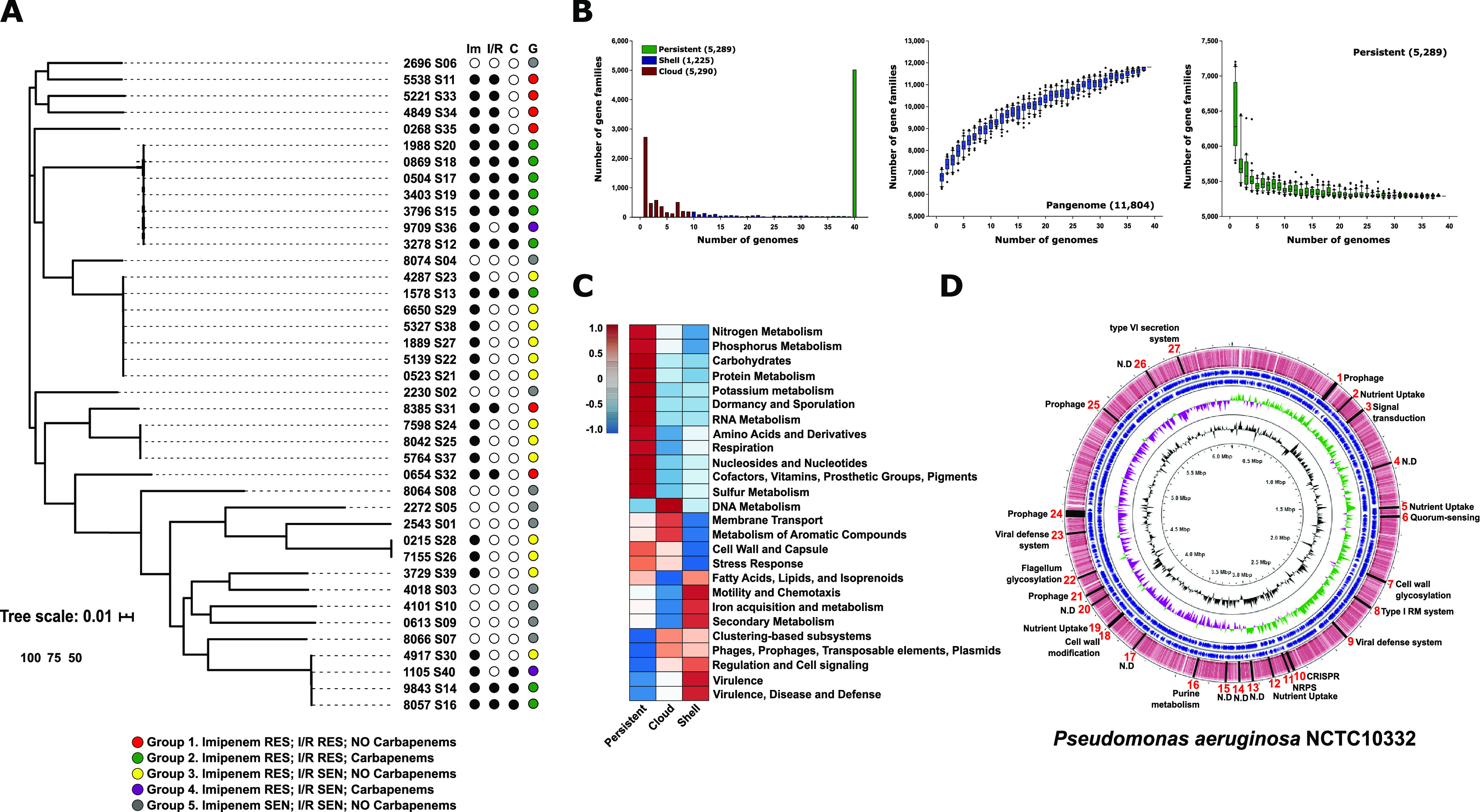
(A) Maximum likelihood phylogenomic tree of the P. aeruginosa genomes isolated in this study. (B) Pangenome and core genome size accumulation, as well as distribution of gene families among the 40 P. aeruginosa strains. (C) Graphical representation of the inferred metabolism with SEED for the different partitions of the pangenome. (D) A schematic representation of the regions of genome plasticity using the P. aeruginosa NCTC10332 genome as a reference.

First, we sought to analyze the genomic diversity of P. aeruginosa strains recovered. We performed a whole-genome phylogenomic tree and average nucleotide identity (ANI) using genomes obtained in this study together with 227 P. aeruginosa reference genomes from a wide range of geographical and isolation sources (see Table S2 in the supplemental material). The results revealed that strains clustered into two groups with ANI values of ca. 98% (Fig. 1A, see Fig. S1 and S2 and Table S4 in the supplemental material), consistent with the cutoff accepted to designate members of same species (15). However, none of our strains represented an independent clade, and we did not find any significant relationship between phylogeny, antibiotic resistance patterns, the origin of isolation, or virulence (Fig. 1A, Fig. S1 and S2, and Table S2). Although all clinical strains are capable of producing similar symptomatology, they are all distributed in independent clades, suggesting a high intrinsic capacity for pathogenesis.

10.1128/mSphere.00836-21.1 FIG S1 Pairwise comparison among P. aeruginosa genomes using both average nucleotide identities (ANIs). Download FIG S1, PDF file, 0.04 MB. Copyright © 2021 López-Pérez et al. 2021 López-Pérez et al. https://creativecommons.org/licenses/by/4.0/ This content is distributed under the terms of the Creative Commons Attribution 4.0 International license.

10.1128/mSphere.00836-21.2 FIG S2 Phylogenomic analysis of P. aeruginosa genomes obtained in this study together with 227 P. aeruginosa reference genomes from the Pseudomonas Genome Database (https://www.pseudomonas.com/) FIG S2, PDF file, 0.2 MB. Copyright © 2021 López-Pérez et al. 2021 López-Pérez et al. https://creativecommons.org/licenses/by/4.0/ This content is distributed under the terms of the Creative Commons Attribution 4.0 International license.

10.1128/mSphere.00836-21.7 TABLE S2 Detailed information about the reference genomes used in this study obtained from the Pseudomonas Genome Database. Download Table S2, XLSX file, 0.03 MB. Copyright © 2021 López-Pérez et al. 2021 López-Pérez et al. https://creativecommons.org/licenses/by/4.0/ This content is distributed under the terms of the Creative Commons Attribution 4.0 International license.

10.1128/mSphere.00836-21.9 TABLE S4 Pairwise comparison among the Pseudomonas aeruginosa genomes using average nucleotide identity (ANI). Download Table S4, XLSX file, 0.9 MB. Copyright © 2021 López-Pérez et al. 2021 López-Pérez et al. https://creativecommons.org/licenses/by/4.0/ This content is distributed under the terms of the Creative Commons Attribution 4.0 International license.

### Pseudomonas aeruginosa pangenome.

We performed a pangenome analysis to evaluate the complete genomic diversity of the strains. While the persistent genome, i.e., number of gene families that are present in almost all the genomes (at least 95%), rapidly reached the plateau, the pangenome curve had not saturated, indicating an open pangenome (Fig. 1B). The persistent genome was 5,289 gene families which represent on average 85.2% of a P. aeruginosa genome. This value also constituted ca. 45% of the entire pangenome (11,804 gene families) (Fig. 1B). The number of genes families that formed the shell genome (at least 15% but less than <95% of the strains) was 1,225 gene families representing 10% of the total pangenome. Finally, gene families presented in less than 15% of strains were classified as the cloud genome (5,290 gene families, comprising ca. 45% of the pangenome). Typically, each new isolate added ca. 380 new gene families.

The gene families were compared against the SEED subsystems database (16) for functional characterization. The fraction of gene families of the persistent genome that could be assigned to a SEED category was 58%, while for the shell and the cloud it was only 28 and 22%, respectively. Both (shell and cloud genome) form the flexible genome, which is related to adaptation to different niches, acquisition of different metabolic capabilities, or even pathogenesis, highlighting the great ignorance we still have about these microbes.

As might be expected, the persistent genome was enriched in categories related to central metabolic processes, such as amino acid biosynthesis, transcription, transduction, and replication (Fig. 1C). The shell and cloud genome shared several categories, such as the presence of prophages and transposable elements or resistance to antibiotics (beta-lactamase) and other compounds, such as mercury, metals, and copper, within the category “virulence, disease, and defense.” Specifically, the cloud genome was enriched in phage defense systems, such as CRISPR systems (“clustering-based subsystems”), toxin-antitoxin systems (“regulation and cell signaling”) or restriction-modification systems (“DNA metabolism”), and protection against oxidative stress (“stress response”), as well as Ton and Tol transport systems and type IV secretion systems within the “membrane transport” category (Fig. 1C). Shell partition was enriched in siderophores (“iron acquisition and metabolism”) (Fig. 1C).

Gene families comprising the flexible genome are often grouped into hot spot regions through the chromosome. Based on the pangenome graphs, we have used the panRGP method (17) to determine these regions of genome plasticity (RGP) using the P. aeruginosa NCTC10332 genome as a reference. We found 27 RGP representing 326 genes (Fig. 1D). It is noteworthy that 11 of the 27 RGP which represent 56.4% of the genes are related to phage-host interactions. We found four prophages as well as viral defense systems, such as CRISPR, restriction-modification systems, and glycosylation islands of the flagellum and the outer membrane which are target structures used by phages (18). Therefore, this interaction can be an important factor in the population dynamics and evolution of these microbes increasing genetic diversity, which could also have an impact on pathogenesis. For this reason, we identified prophage sequences based on the PHASTER prediction (19) in all the P. aeruginosa strains and functionally annotated all those genes. Three of these sequences were related to antibiotic resistance families such as class B beta-lactamases, aminoglycoside *O*-nucleotidyltransferases, and major facilitator superfamily efflux pumps. Interestingly, one of the sequences was annotated as a zonula occludens toxin, an enterotoxin described in Vibrio cholerae that increases mucosal permeability (20).

### Pseudomonas aeruginosa mobilome.

Given that most of the virulence and antibiotic resistance factors are present in the flexible genome (cloud + shell genome), we decided to analyze the impact of DNA fragment transfer by analyzing recombination as well as the MGEs involved in its dispersion.

Using the core genome alignment of all the isolates, we computed the relative rate of recombination to mutation (γ/μ) using mcorr (21), which was estimated to be 8.9 (standard deviation [SD] of 0.525). These data suggest that recombination produces more nucleotide replacements than mutations. A comparison with two other important Gram-negative nosocomial pathogens (Klebsiella pneumoniae and Acinetobacter baumannii) revealed that P. aeruginosa has much higher recombination values. Using the same methodology, K. pneumoniae and A. baumannii had γ/μ values of 4.2 and 1.3, respectively (21). However, *P. aeruginosa* showed lower values than *Mycobacterium abscessus*, another opportunistic pathogen that had the highest recombination values (γ/μ = 13) (21).

**FIG 2:**
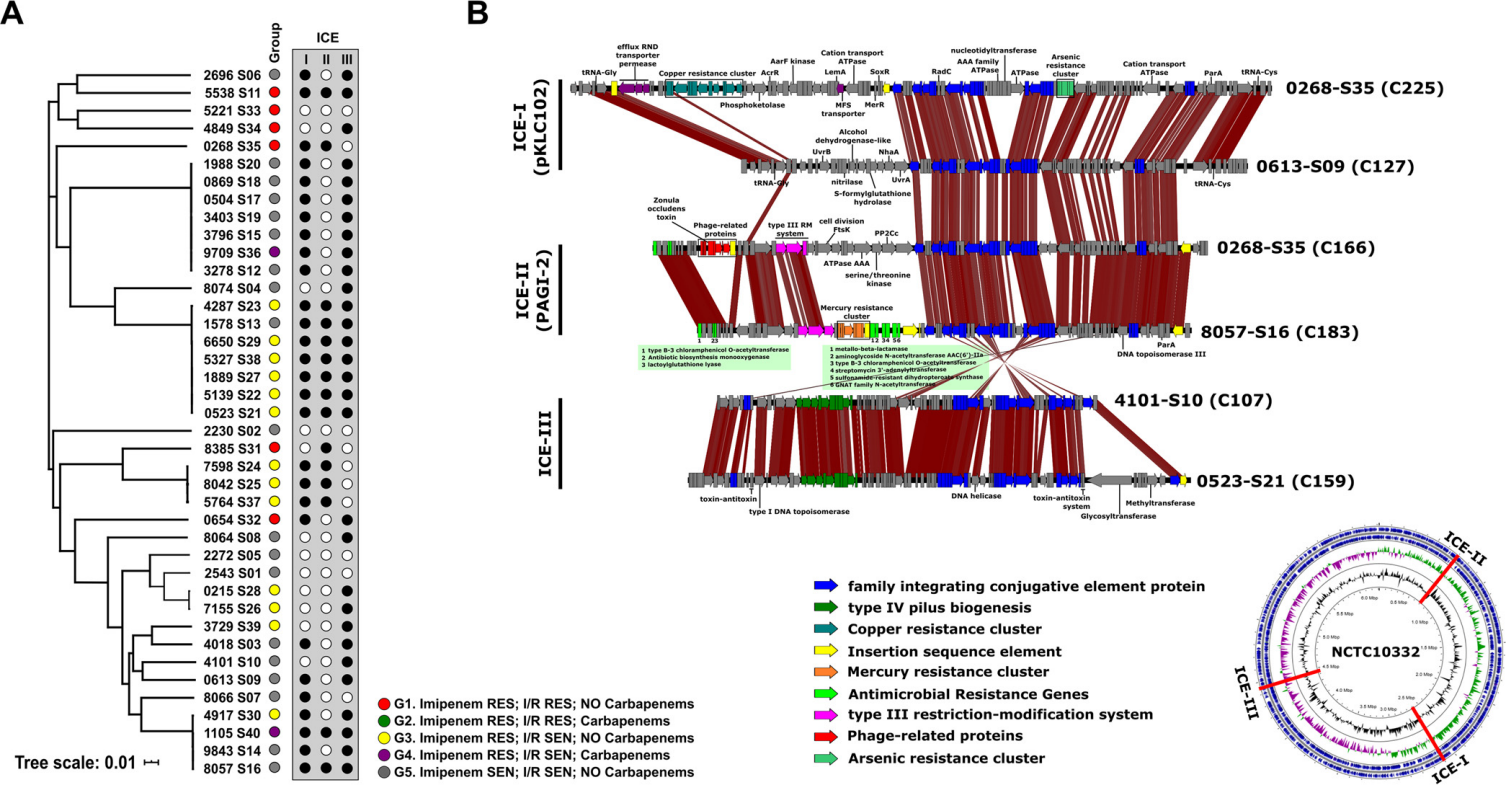
(A) Maximum likelihood phylogenomic tree of the P. aeruginosa genomes isolated in this study; the box on the right shows the type of ICE present in each of the genomes. (B) Comparison of the gene content of the ICE in the strain genomes. Predicted open reading frames (ORFs) with the same color are involved in the same function. The genomic map indicates the location of the three types of ICEs in the reference genome (P. aeruginosa NCTC10332).

The genomic comparison revealed the presence of three different types of integrative and conjugative elements (ICEs) (22). We found this type of MGE in 90% of the strains (36 out of 40), and all of them belonged to three different types (Fig. 2A). The first one corresponding to the pKLC102 family was inserted in a tRNA-Gly. Cargo genes from this region were related to niche adaptation, such as resistance to different environmental compounds (copper, chromate, arsenic, and mercury), as well as an enrichment in LysR transcriptional regulators that can be beneficial for the host cell (23) (Fig. 2B). The second type stood out for having restriction systems for protection against phages but mainly for the presence of two clusters with multiple genes of resistance to antibiotics. The first antibiotic cluster was associated with a Tn*3*-like transposon present in a group of three (1105-S40, 9843-S14, and 8057-S16) of four strains with an ANI of >99% which indicates the ease of movement of these elements among strains (Fig. 2B). The antibiotic resistance genes located in this cluster were VIM beta-lactamases, an aminoglycoside *N*-acetyltransferase AAC(6′)-IIa, a type B-3 chloramphenicol *O*-acetyltransferase, a streptomycin 3′-adenylyltransferase, a sulfonamide-resistant dihydropteroate synthase, and a Gcn5-related *N*-acetyltransferases (Fig. 2B). On the other hand, the second cluster had three genes that confer resistance to chloramphenicol, fosfomycin, and aminoglycoside antibiotics. Furthermore, within the 0268-S35 strain, we also found a defective prophage with a sequence encoding the zonula occludens toxin. The third type of ICE showed only a small variable region among the strains, which mostly encodes several toxin-antitoxin systems (Fig. 2B).

### Antimicrobial genes.

Next, we analyzed the presence of antimicrobial resistance genes (ARGs) using the MEGARes 2.0 database (24). These predictions were correlated with their correspondence in the different pangenome partitions. Using a 70% threshold (BLASTP), a total of 154 ARGs were detected as grouping into 28 families. The most represented families were “drug and biocide resistance” (21.4%), including different types of efflux pumps and regulators (resistance-nodulation-division [RND], ATP-binding cassette [ABC], and multidrug and toxic compound extrusion [MATE] transporters); “mercury resistance” (12.3%); “multimetal resistance” (9.1%); “beta-lactams” (8.4%); and “copper resistance” (5.8%). The persistent genome contains about 54% of the ARGs and is where most of the efflux pumps and regulators are concentrated. This finding highlights the intrinsic capacity of these microbes for antimicrobial resistance. In the other two partitions, one-half of the ARGs were related to resistance to mercury, beta-lactams, and aminoglycosides in the cloud genome (ca. 29% of total ARGs) and resistance to mercury, copper, and beta-lactams in the shell genome (ca. 17% of total ARGs). The analysis of the genomic context where the ARGs were located in the flexible genome revealed that 44% were associated with ICEs (including associated transposons), while 17% were located in additive islands and only 3 sequences in plasmids. Interestingly, antibiotic resistance genes showed a tendency to cluster in the same array within the ICEs conferring multiple resistance which increases the risk to human health. The rest of the sequences were found in contigs too small to be able to infer whether they were associated with a specific mobile genetic element.

### Imipenem-relebactam resistance with carbapenemases.

Through comparative genomics, we evaluated the dynamics and mechanisms that these strains have developed to acquire resistance to I/R. We found six genomes belonging to the G3 and one genome from G2, strain 1578-S13, that clustered together within the same clonal frame (ANI , >99%) (Fig. 1A and S1). These differences allowed us to study specific genomic differences that drive the phenotypic differences by subtracting the common part of both pangenomes. We found 119 specific genes in the 1578-S13 genome. Twelve genes were concentrated in a contig of only 8 kb. Among others, a gene coding for an AAC(6′)-Iag, an aminoglycoside acetyltransferase related to aminoglycoside antibiotic resistance, and a VIM-4 (carbapenemases class B), which could putatively evade beta-lactam inhibitors like relebactam, were found. Next to these genes, we also found transposition genes, a TniA and TniQ module, which are normally associated with a part of class 1 integrons and are derivatives of Tn*402* (also called Tn*5090*) (25).

A BLAST search of the VIM-4 gene sequence against the nonredundant (nr) NCBI database showed that this gene was found in a plethora of MGEs, such as integrons, plasmids, or ICEs, in clinical strains isolated worldwide within members of *Pseudomonadales*, *Burkholderiales*, and *Enterobacterales* orders (see Fig. S3 in the supplemental material). Therefore, the presence of orthologs of this gene in different mobile elements of different taxonomic ranges multiplies the risk of increasing resistance to the I/R combination in a short period of time.

10.1128/mSphere.00836-21.3 FIG S3 Genomic alignment of the mobile genetic elements containing the ortholog gene to VIM-4 found within members of *Pseudomonadales*, *Burkholderiales*, and *Enterobacterales* orders. Download FIG S3, PDF file, 0.04 MB. Copyright © 2021 López-Pérez et al. 2021 López-Pérez et al. https://creativecommons.org/licenses/by/4.0/ This content is distributed under the terms of the Creative Commons Attribution 4.0 International license.

### Imipenem-relebactam resistance without carbapenemases.

To elucidate genetic factors associated with the resistance to I/R, we compared genomes from G1 against those from G3 (Fig. 1). We subtracted the common part of the pangenomes from both groups to analyze those gene families unique to G3 and obtained a total of 1,174 genes families. However, an analysis of the prevalence of these families among the G3 genomes showed that none of them was found in more than one genome. This result could suggest that (i) there is a wide diversity of mechanisms involved in resistance and (ii) the resistance mechanism could be located in the persistent genome. In the latter case, the mutation of some gene or noncoding region could lead to the modification of its expression. Therefore, we decided to analyze the microdiversity between resistant and sensitive strains to detect possible target genes.

### Imipenem-relebactam resistance (microdiversity).

**FIG 3:**
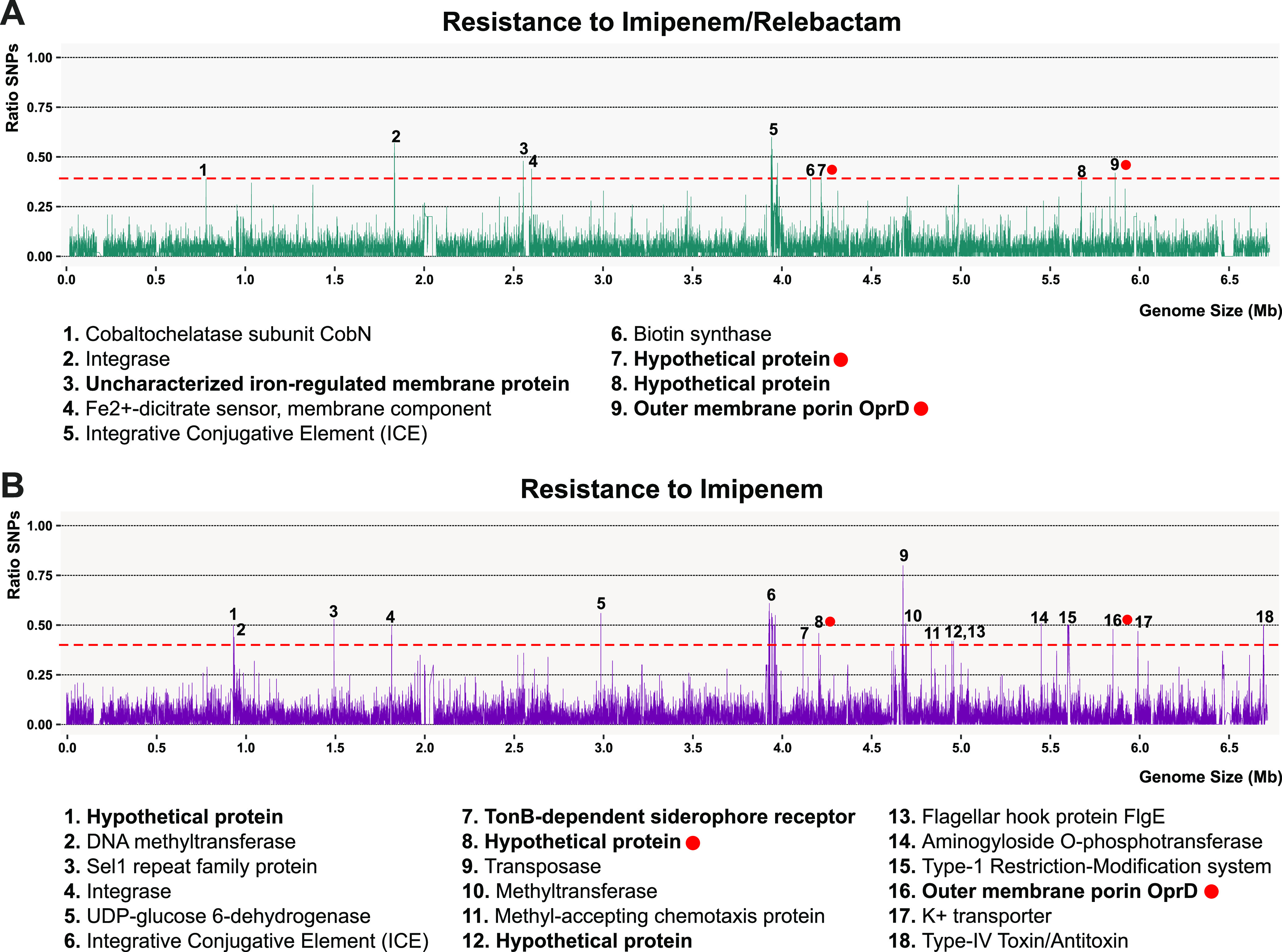
Distribution of single-nucleotide polymorphisms (SNPs) along the genome of 2696_S6. SNP frequency considering several strains resistant to imipenem-relebactam (5 strains) (A) and imipenem (11 strains) (B). Numbered peaks are annotated on the bottom. In bold, proteins with at least one membrane domain. Red dots indicated shared proteins between treatments.

We next sought to determine whether the resistance to carbapenems is due to the presence of single-nucleotide polymorphisms (SNPs). Therefore, we grouped 7 isolates susceptible to Im and compared them to 11 and 6 isolates resistant to Im and I/R, respectively. Only isolates with clear evidence to not harbor carbapenemases were considered. For a given position, we considered all the possible nucleotides present in the susceptible group as nonsignificant mutations through a window of 100 nucleotides and significant those SNPs present in the resistant groups and different from the susceptible one (see Materials and Methods). In the end, we could retrieve several signals of high nucleotide variations for Im and I/R on which more than 40% of the strains in these groups have SNPs (Fig. 3). Due to high recombination detected, we found SNPs in common proteins, such as integrases, transposases, methyltransferases, and restriction-modification systems, and along the ICE. Remarkably, seven proteins carrying membrane domains, mostly hypothetical, had also a high proportion of SNPs. We found among these proteins the outer membrane porin OprD in both antibiotics. This protein participates in the passive uptake of basic amino acids across the outer membrane, but it is also permeable to carbapenems (26). By real-time PCR and protein detection, previous studies demonstrated that the low-to-absent expression of the *oprD* gene is frequently noted in carbapenem-resistant isolates without carbapenemase activity (27). We performed the alignment of the OprD protein among susceptible and resistant isolates to identify those possible mutations that would confer resistance to Im (see Fig. S4 in the supplemental material). In the end, a total of 103 and 32 synonymous and nonsynonymous mutations, respectively, were detected along this gene. It is important to highlight that 81% of the resistant strains had mutations that produced a premature stop codon, regardless of the presence of relebactam, as expected given that the compound acts as a beta-lactamase inhibitor (28). On the other hand, three resistant isolates carried the full protein. However, due to the amino acid variability within the susceptible group, we could not determine any significant mutation that might correlate with their resistance to Im; thus, other molecular mechanisms may take place. Our results agree with previous reports that indicated that truncated OprD proteins are responsible for the resistance to Im (29).

10.1128/mSphere.00836-21.4 FIG S4 Amino acid alignment of OprD (443 amino acids [aa]) among susceptible (in blue) and resistant strains of P. aeruginosa to imipenem (pink) and imipenem-relebactam (red). Only nonsynonymous mutations are shown. Green and black letters indicate whether the amino acid is the same as the reference strain (2696-S6). Stars indicate the stop of the protein. Download FIG S4, PDF file, 0.1 MB. Copyright © 2021 López-Pérez et al. 2021 López-Pérez et al. https://creativecommons.org/licenses/by/4.0/ This content is distributed under the terms of the Creative Commons Attribution 4.0 International license.

Finally, the same methodology was applied for the comparison between Im versus I/R resistant strains. Considering the same threshold of 40% of the strains having a SNP in a given position, we detected 54 hot spots of SNP accumulation. Nine peaks corresponded to intergenic spacers, whereas the remaining 46 peaks were found in coding sequences, such as the flagellar biosynthesis operon, type VI secretion proteins, nonribosomal peptide-synthetase (NRPS) systems, and a few membrane proteins, which were mostly transporters related to iron (signals 1, 27, and 30), cyanate (9), and lipoprotein (38) (see Fig. S5 in the supplemental material). Besides, seven signals were found in proteins, and their functions have not been characterized yet. The high divergence between strains leads to a large accumulation of mutations which makes it difficult to determine an exact target associated with resistance. However, the analysis revealed several transporters or porins that should be studied in detail by knockout. In addition, the development of *in vitro* resistant mutants would be a good approach to complement the results obtained.

10.1128/mSphere.00836-21.5 FIG S5 Distribution of single-nucleotide polymorphisms (SNPs) along the genome of 7598-S24, resistant to imipenem. SNP frequency considering several strains resistant to imipenem-relebactam (5 strains). Numbered peaks are annotated on the right. In bold, proteins with at least one membrane domain. Download FIG S5, PDF file, 0.2 MB. Copyright © 2021 López-Pérez et al. 2021 López-Pérez et al. https://creativecommons.org/licenses/by/4.0/ This content is distributed under the terms of the Creative Commons Attribution 4.0 International license.

The data suggest that both the flexible and persistent (or core) genome are closely related to I/R resistance. This result, together with the high metabolic flexibility due to the large number of transcriptional regulators that control the expression of the secretion or quorum sensing systems, make it possible for these microbes to adapt rapidly to environmental stresses, such as antibiotics (30). However, the analysis of mutations or presence/absence of genes did not yield a clear result, which could suggest that the processes leading to I/R resistance are multifactorial or that regulation occurs at the transcriptional level exerting its effect on gene expression. Thus, detection by traditional PCR-based means would not be feasible. Future studies should be directed to transcriptional analysis between sensitive and resistant strains.

The high recombination and the concentration of multiple resistances in small clusters of genes easily transmitted mainly by ICEs make the therapeutic options in the fight against antibiotic resistance increasingly limited. Due to the urgency and seriousness of the problem, we must anticipate the emergence of threats that may arise, which is why the implementation of genomics in the hospital environment is a crucial point in the fight against resistance. Analyzing genomic diversity as well as tracking new genes responsible for resistance is crucial for designing more accurate variant detection and monitoring strategies. Overall, this study not only advances the knowledge of genetic diversity in these strains but also offers a methodology that can apply to many other opportunistic pathogens with broad antibiotic resistance, such as A. baumannii or K. pneumoniae.

## MATERIALS AND METHODS

### P. aeruginosa isolation and sequencing.

Strains were collected during 2020 from clinical isolates of patients hospitalized at Hospital General Universitario de Alicante (Spain). Antimicrobial susceptibility test was carried out with the MicroScan WalkAway system (Beckman). The origin of the isolates and their antibiotic resistance patterns are detailed in Table S1. MALDI-TOF mass spectrometry was used for species identification. The presence of carbapenemases was determined using the Gene Xpert system (Cepheid). DNA was extracted using Chelex 100 Resin (Bio-Rad) and checked for quality on a 1% agarose gel. Sequencing was performed using the Illumina HiSeq 2000 (100-bp paired-end reads) platform.

### Genome comparison and phylogeny.

The generated reads were trimmed and assembled using Trimmomatic v0.36 (31) and SPAdes v3.11.1 (32), respectively. The resulting genes on the assembled contigs were predicted using Prodigal v2.6 (33). tRNA genes were predicted using tRNAscan‐SE v1.4 (34) and ssu‐align v0.1.1 (35) along with meta‐rna (36) for rRNA genes. Predicted protein sequences were compared against the NCBI nr database using DIAMOND (37) and against COG (38) and TIGRFAMs (39) using HMMscan v3.1b2 (40) for taxonomic and functional annotation. AMRs were detected in our samples using the MEGARes 2.0 database (24). Assembled proteins were aligned to the reference database using DIAMOND (≥50% identity, ≥50% alignment length, E value of <10^−5^). Average nucleotide identity (ANI) and coverage between pairs of genomes were calculated using the PYANI software (41). We applied the software mcorr (21) to infer the parameters of homologous recombination, i.e., the rate of recombination to mutation (*γ/μ*). To classify the strains phylogenomically, genomes were analyzed using TIGRFAMs to identify and concatenate all the conserved proteins. The concatenated proteins were aligned using Kalign (42), and a maximum likelihood tree was made using FastTree (43) using a JTT + CAT model and a gamma approximation. As a reference, all available genomes belonging to the species Pseudomonas aeruginosa were downloaded from the Pseudomonas Genome Database (https://www.pseudomonas.com/) (Table S2).

### Pangenome analysis.

Pangenomes were generated using PPanGGOLiN software, and gene families were divided into persistent/shell/cloud partitions (44). Then, the SEED subsystem database was used to determine the functional annotation of genes that constituted each partition (16). The comparison was made using DIAMOND (37), keeping all matches with an E value of <0.001 and alignment length of >0.5 for both subject and query. The panRGP method (17) was used to predict regions of genome plasticity (RGPs) using pangenome graphs made of all available genomes obtained from PPanGGOLiN analysis.

### Identification of single-nucleotide polymorphisms (SNPs).

SNPs were determined by aligning trimmed Illumina reads to the reference genomes using Bowtie 2 (45). Only alignments with an error rate of <0.1% were considered. Variants were then detected using Varscan (46), considering that, for a given polymorphism, it had to be present in at least 80% of the aligned reads. Lastly, SnpEff (47) was used to discriminate between synonymous, nonsynonymous, and intergenic mutations. We called a mutation only if the SNP was different from those detected in the susceptible group (e.g., A and G variations in the susceptible group, only T and C are called mutations in the resistant strain). Only isolates with clear evidence to not harbor carbapenemases were analyzed. Finally, the amino acid alignment of OprD protein sequences was performed with MUSCLE (48).

### Data availability.

The genomes have been deposited under BioProject PRJNA754264.
