# Supplementary material for: Genomic Characterization of Imipenem- and Imipenem-Relebactam-Resistant Clinical Isolates of Pseudomonas aeruginosa
Source: mSphere. 2021 Nov 24;6(6):e00836-21. doi: 10.1128/mSphere.00836-21 (PMC8612254; doi:10.1128/mSphere.00836-21)
Supplement: FIG S1 [file msphere.00836-21-sf001.pdf]

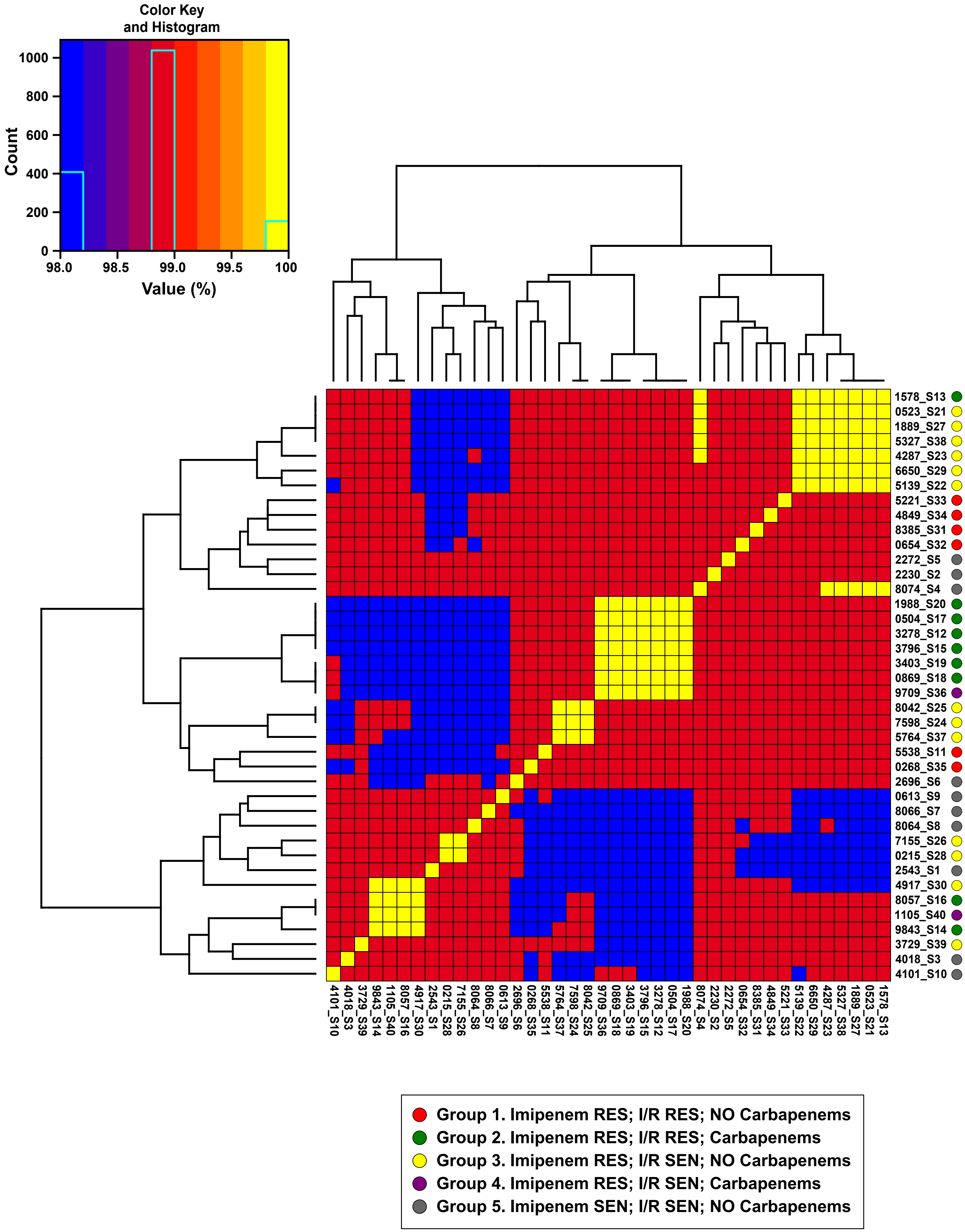

Color Key
and Histogram
Count
0
200
400
600
800
1000
98.0
98.5
99.0
99.5
100
Value (%)
1578_S13
0523_S21
1889_S27
5327_S38
4287_S23
6650_S29
5139_S22
5221_S33
4849_S34
8385_S31
0654_S32
2272_S5
2230_S2
8074_S4
1988_S20
0504_S17
3278_S12
3796_S15
3403_S19
0869_S18
9709_S36
8042_S25
7598_S24
5764_S37
5538_S11
0268_S35
2696_S6
0613_S9
8066_S7
8064_S8
7155_S26
0215_S28
2543_S1
4917_S30
8057_S16
1105_S40
9843_S14
3729_S39
4018_S3
4101_S10
Group 1. Imipenem RES; I/R RES; NO Carbapenems
Group 2. Imipenem RES; I/R RES; Carbapenems
Group 3. Imipenem RES; I/R SEN; NO Carbapenems
Group 4. Imipenem RES; I/R SEN; Carbapenems
Group 5. Imipenem SEN; I/R SEN; NO Carbapenems
