# Supplementary material for: Genomic Characterization of Imipenem- and Imipenem-Relebactam-Resistant Clinical Isolates of Pseudomonas aeruginosa
Source: mSphere. 2021 Nov 24;6(6):e00836-21. doi: 10.1128/mSphere.00836-21 (PMC8612254; doi:10.1128/mSphere.00836-21)
Supplement: FIG S2 [file msphere.00836-21-sf002.pdf]

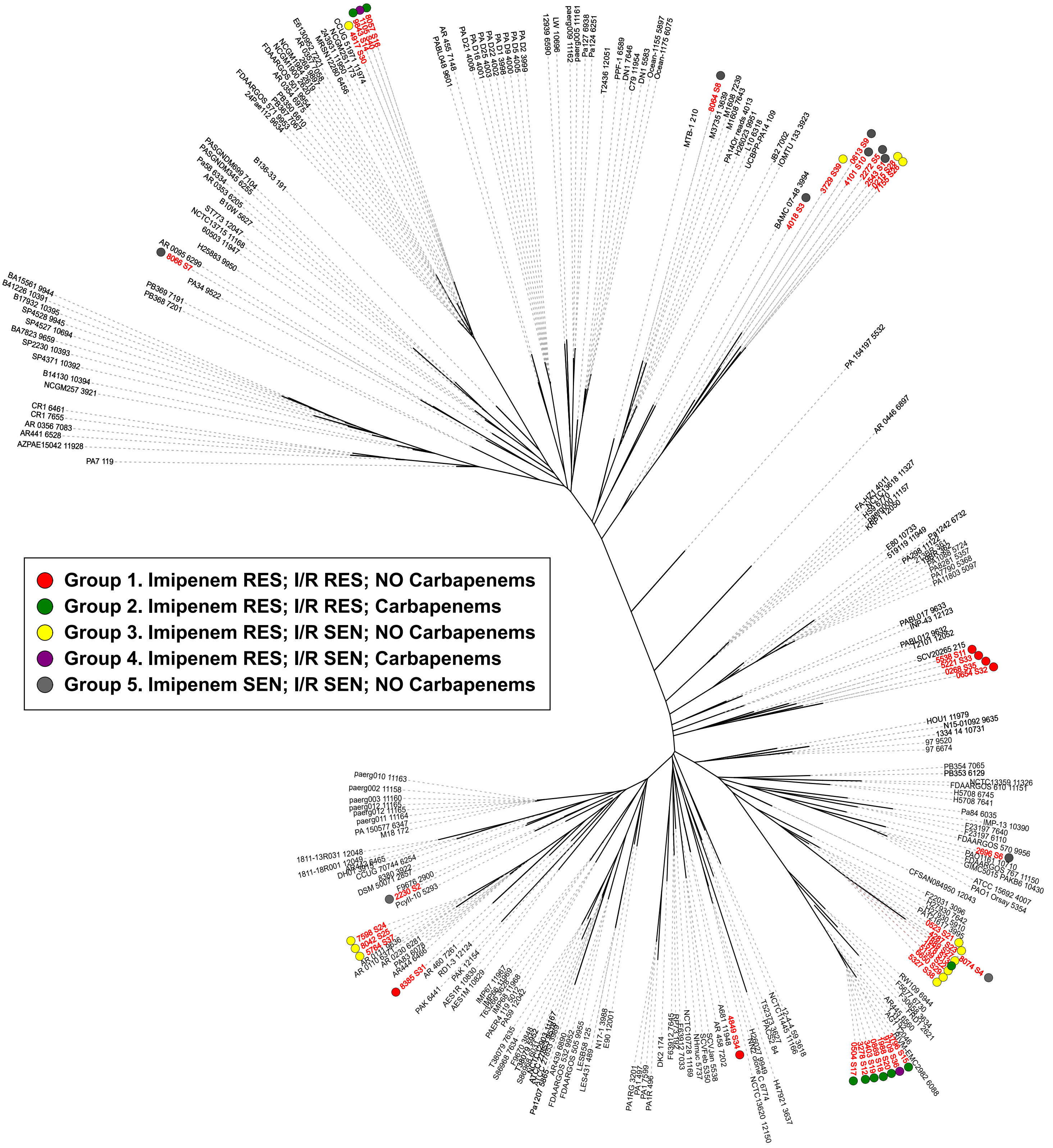

Group 1. Imipenem RES; I/R RES; NO Carbapenems
Group 2. Imipenem RES; I/R RES; Carbapenems
Group 3. Imipenem RES; I/R SEN; NO Carbapenems
Group 4. Imipenem RES; I/R SEN; Carbapenems
Group 5. Imipenem SEN; I/R SEN; NO Carbapenems
BA15561 9944
B41226 10391
B17932 10395
SP4528 9945
SP4527 10694
BA7823 9659
SP2230 10393
SP4371 10392
B14130 10394
NCGM257 3921
CR1 6461
CR1 7655
AR 0356 7083
AR441 6528
AZPAE15042 11928
PA7 119
PB369 7191
PB368 7201
PA34 9522
AR 0095 6299
8066 S7
PA 154197 5532
AR 0446 6897
HOU1 11979
N15-01092 9635
1334 14 10731
97 9520
97 6674
PB354 7065
PB353 6129
paerg010 11163
paerg002 11158
paerg003 11160
paerg011 11164
PA 150577 6347
M18 172
