# Supplementary material for: Genomic Characterization of Imipenem- and Imipenem-Relebactam-Resistant Clinical Isolates of Pseudomonas aeruginosa
Source: mSphere. 2021 Nov 24;6(6):e00836-21. doi: 10.1128/mSphere.00836-21 (PMC8612254; doi:10.1128/mSphere.00836-21)
Supplement: FIG S3 [file msphere.00836-21-sf003.pdf]

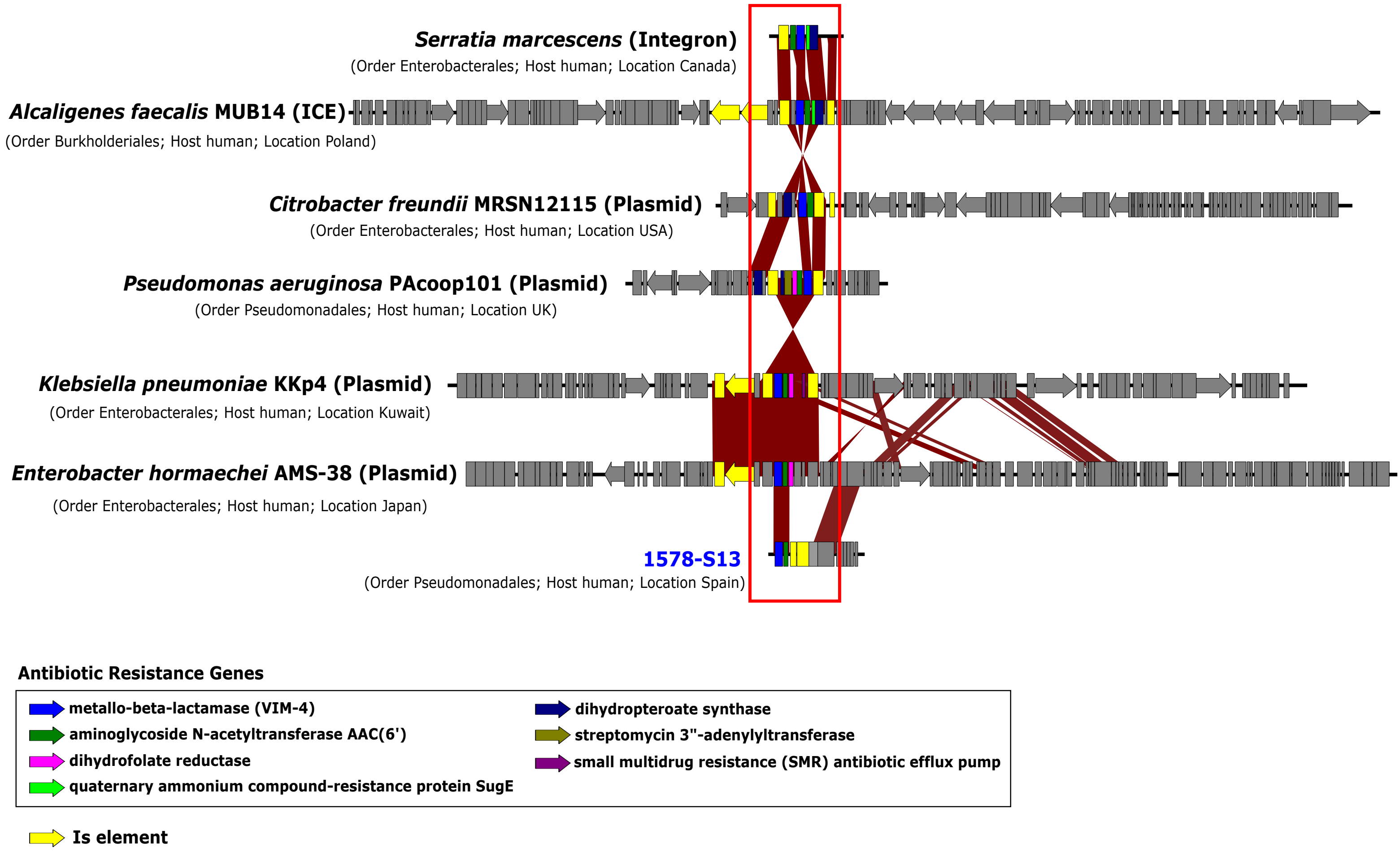

Serratia marcescens (Integron)
(Order Enterobacterales; Host human; Location Canada)
Alcaligenes faecalis MUB14 (ICE)
(Order Burkholderiales; Host human; Location Poland)
Citrobacter freundii MRSN12115 (Plasmid)
(Order Enterobacterales; Host human; Location USA)
Pseudomonas aeruginosa PAcoop101 (Plasmid)
(Order Pseudomonadales; Host human; Location UK)
Klebsiella pneumoniae KKp4 (Plasmid)
(Order Enterobacterales; Host human; Location Kuwait)
Enterobacter hormaechei AMS-38 (Plasmid)
(Order Enterobacterales; Host human; Location Japan)
1578-S13
(Order Pseudomonadales; Host human; Location Spain)
Antibiotic Resistance Genes
metallo-beta-lactamase (VIM-4)
aminoglycoside N-acetyltransferase AAC(6')
dihydrofolate reductase
quaternary ammonium compound-resistance protein SugE
dihydropteroate synthase
streptomycin 3''-adenylyltransferase
small multidrug resistance (SMR) antibiotic efflux pump
Is element
