# Supplementary material for: Genomic Characterization of Imipenem- and Imipenem-Relebactam-Resistant Clinical Isolates of Pseudomonas aeruginosa
Source: mSphere. 2021 Nov 24;6(6):e00836-21. doi: 10.1128/mSphere.00836-21 (PMC8612254; doi:10.1128/mSphere.00836-21)
Supplement: FIG S4 [file msphere.00836-21-sf004.pdf]

	Isolate	-40 -57 -59 -67 -103 -115 -127 -138 -169 -170 -185 -186 -189 -202 -210 -230 -240 -250 -262 -267 -276 -281 -296 -301 -310 -312 -315 -327 -347 -403 -424 -425
	2696-S6 (SEN)	DSSQTKVWEFEPVEIESQNATAKQRGAQLSQG
	2230-S2 (SEN)	DSSQTKVWEFEPVEIESQNATAKQRGAQLSQG
	2272-S5 (SEN)	DERQTKLWEFQGTQAKTQTAAAQEEGGQMAEG
	2543-S1 (SEN)	DSSQSTVWELQGTEIESQNATAKQEGGQLSQA
	8064-S8 (SEN)	DSSQSTVWELQGTEIESQNATAKQEGGQLSQA
	8066-S7 (SEN)	DSSQSTVWELQGTEIESQNATAKQEGGQLSQA
	8074-S4 (SEN)	DSSQTKLWEFQGTQAKTQTAAAQERGAQMAEG
	0215-S28 (RES I)	DSSQTKLWEFQGTQAKTQTAAGQEERGQMAEG
	7155-S26 (RES I)	DSSQTKLWEFQGTQAKTQTAAGQEERGQMAEG
	5538-S11 (RES IR)	DSSQSTVWELEPVEIESQNATAQQRGAQLSQG
Truncated proteins	0523-S21 (RES I)	NERQTKVWEFEPVQAKTQTSTGQEGGA*
	1889-S27 (RES I)	NERQTKVWEFEPVQAKTQTSTGQEGGA*
	5139-S22 (RES I)	NERQTKVWEFEPVQAKTQTSTGQEGGA*
	5327-S38 (RES I)	NERQTKVWEFEPVQAKTQTSTGQEGGA*
	6650-S29 (RES I)	NERQTKVWEFEPVQAKTQTSTGQEGGA*
	4849-S34 (RES IR)	NERQTKVWEFEPVQAKTQTSTGQEGGA*
	5221-S33 (RES IR)	NERQTKVWEFEPVQAKTQTSTGQEGGA*
	8385-S31 (RES IR)	DERQTKVWEFEPVQAKTQTSTGQEGGA*
	0268-S35 (RES IR)	NERYTKVWEFEPVQAKT*
	5764-S37 (RES I)	NERQTKVW*
	7598-S24 (RES I)	NERQTKVW*
	8042-S25 (RES I)	NERQTKVW*
	4287-S23 (RES I)	NERQTKV**
