# Supplementary material for: Genomic Characterization of Imipenem- and Imipenem-Relebactam-Resistant Clinical Isolates of Pseudomonas aeruginosa
Source: mSphere. 2021 Nov 24;6(6):e00836-21. doi: 10.1128/mSphere.00836-21 (PMC8612254; doi:10.1128/mSphere.00836-21)
Supplement: FIG S5 [file msphere.00836-21-sf005.pdf]

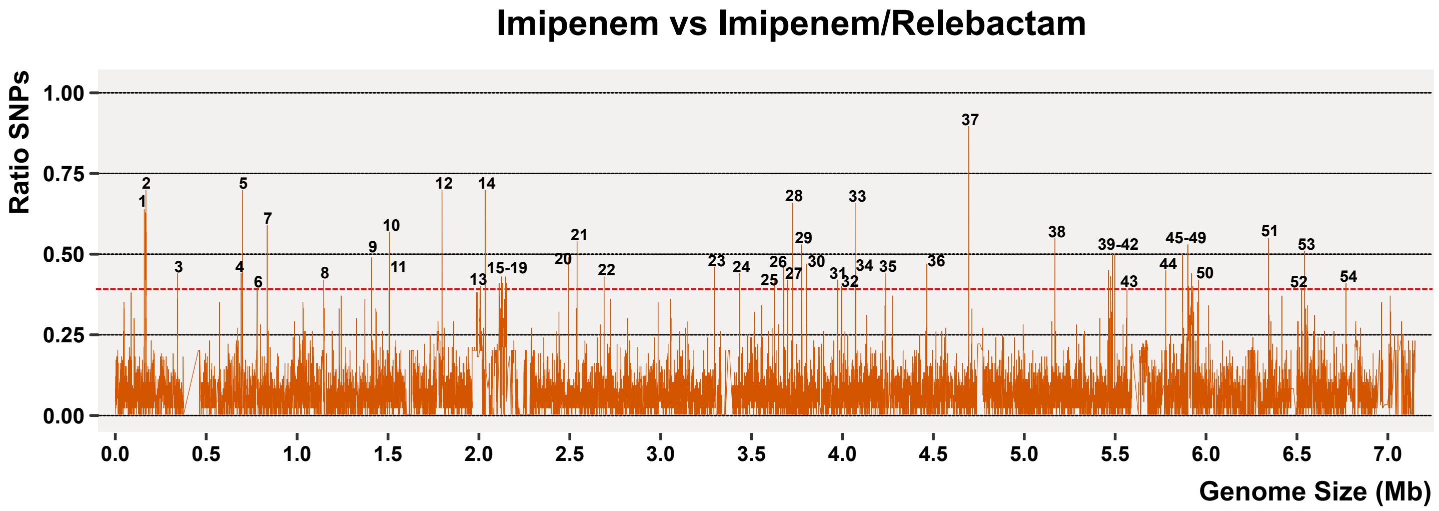

1. Acetyltransferase
2. TonB-dependent siderophore receptor
3. Amino acid adenylation domain protein
4. ADP-ribosyltransferase
5. *Intergenic spacer*
6. AraC transcriptional regulator
7. *Intergenic spacer*
8. Alcohol dehydrogenase
9. MFS transporter
10. Diguanylate cyclase/phosphodiesterase
11. RraB-like superfamily
12. N-acetyltransferase
13. Conjugal transfer protein TrbE
14. *Intergenic spacer*
15. Integrase
16. RepA replication protein
17. RepA replication protein
18. ATPase
19. Hypothetical protein
20. *Intergenic spacer*
21. Glycosyltransferase
22. 16S rRNA (cytosine(967)-C(5))-methyltransferase RsmB
23. Disulfide bond formation protein DsbB
24. *Intergenic spacer*
25. *Intergenic spacer*
26. *Intergenic spacer*
27. TonB-dependent receptor
28. Hyphothetical protein
29. Endonuclease/exonuclease/phosphatase
30. Low affinity iron permease
31. *Intergenic spacer*
32. Hyphothetical protein
33. LysR family transcriptional regulator
34. Short-chain dehydrogenase
35. transcriptional regulator
36. ATPase, type IV, pilus assembly, PilB
37. Methyl-accepting chemotaxis protein
38. Lipoprotein-releasing system transmembrane protein
39. Hyphothetical protein
40. Hyphothetical protein
14. Flagellar hook-associated protein FlgK
15. Flagellin
43. Hyphothetical protein
44. *Intergenic spacer*
45. Type VI secretion system
46. Cyclic peptide transporter
47. TonB-dependent siderophore receptor
48. Non-ribosomal peptide synthetase
49. Non-ribosomal peptide synthetase
50. Outer membrane usher protein
51. Acyl-CoA transferase
52. Hemolysin activation/secretion protein
53. Alkaline phosphatase L
54. Excinuclease ABC subunit A
